# Evaluation of lower extremity gait analysis using Kinect V2^®^ tracking system

**DOI:** 10.1051/sicotj/2022027

**Published:** 2022-06-24

**Authors:** Takuya Usami, Kazuki Nishida, Hirotaka Iguchi, Taro Okumura, Hiroaki Sakai, Ruido Ida, Mitsuya Horiba, Shuuto Kashima, Kento Sahashi, Hayato Asai, Yuko Nagaya, Hideki Murakami, Yoshino Ueki, Gen Kuroyanagi

**Affiliations:** 1 Department of Orthopedic Surgery, Nagoya City University Graduate School of Medical Sciences Nagoya Aichi 467-8601 Japan; 2 Nagoya City University East Medical Center Nagoya Aichi 464-8547 Japan; 3 Center for Advanced Medicine and Clinical Research Nagoya University Hospital Nagoya Aichi 466-8560 Japan; 4 National Hospital Organization Toyohashi Medical Center Nagoya Aichi 440-8510 Japan; 5 Department of Rehabilitation Medicine, Nagoya City University Graduate School of Medical Sciences Nagoya Aichi 467-8601 Japan

**Keywords:** Gait analysis, Kinect V2, Motion capture system, Posture recognition, Skeleton definition

## Abstract

*Introduction*: Microsoft Kinect V2^®^ (Kinect) is a peripheral device of Xbox^®^ and acquires information such as depth, posture, and skeleton definition. In this study, we investigated whether Kinect can be used for human gait analysis. *Methods*: Ten healthy volunteers walked 20 trials, and each walk was recorded by a Kinect and infrared- and marker-based-motion capture system. Pearson’s correlation and overall agreement with a method of meta-analysis of Pearson’s correlation coefficient were used to assess the reliability of each parameter, including gait velocity, gait cycle time, step length, hip and knee joint angle, ground contact time of foot, and max ankle velocity. Hip and knee angles in one gait cycle were calculated in Kinect and motion capture groups. *Results*: The coefficients of correlation for gait velocity (*r* = 0.92), step length (*r* = 0.81) were regarded as strong reliability. Gait cycle time (*r* = 0.65), minimum flexion angle of hip joint (*r* = 0.68) were regarded as moderate reliability. The maximum flexion angle of the hip joint (*r* = 0.43) and maximum flexion angle of the knee joint (*r* = 0.54) were regarded as fair reliability. Minimum flexion angle of knee joint (*r* = 0.23), ground contact time of foot (*r* = 0.23), and maximum ankle velocity (*r* = 0.22) were regarded as poor reliability. The method of meta-analysis revealed that participants with small hip and knee flexion angles tended to have poor correlations in maximum flexion angle of hip and knee joints. Similar trajectories of hip and knee angles were observed in Kinect and motion capture groups. *Conclusions*: Our results strongly suggest that Kinect could be a reliable device for evaluating gait parameters, including gait velocity, gait cycle time, step length, minimum flexion angle of the hip joint, and maximum flexion angle of the knee joint.

## Introduction

Gait analysis provides kinematic changes in joints during gait, providing a better understanding of changes in joint function and pathological conditions. The movement of joints during walking in patients with orthopedic joint disease is different from that in healthy individuals. On the other hand, various factors such as implant alignment and surgical method are known to affect postoperative clinical outcomes after lower extremity joint surgeries [1, 2]. Gait velocity and range of motion of the knee and hip joints are normally recovered after surgery [3, 4]. However, how other kinematic or spatiotemporal parameters affect clinical outcomes has not been fully clarified.

Several technologies, including video examination, force plate examination, electromyography, accelerometer, and infrared-based-motion capture, have been developed to evaluate human gait parameters [5–7]. Among them, infrared- and marker-based-motion capture systems such as Optorack^®^ (Northern Digital Inc., ON, Canada) and VICON^®^ (Vicon Motion Systems, Oxford, UK), are widely used for gait analysis. These systems show high reliability of kinematic parameters, including position, velocity, and acceleration [7–9]. However, these systems necessitate multiple cameras and a large amount of space, and the equipment is expensive. In this study, we investigated whether Microsoft Kinect V2^®^, which is a low-cost, portable and peripheral device of Xbox^®^, evaluates kinematic and spatiotemporal parameters and can be used for human gait analysis in healthy individuals.

## Materials and method

### Participant

From June 2020 to December 2020, 10 young, healthy volunteers (one male and nine females) were enrolled in this study. Informed consent was obtained firmly from each participant. Mean height and body weight was 169.1 ± 7.0 cm and 59.2 ± 6.6 kg, respectively (Table 1). To minimize measurement errors, 10 walks were captured simultaneously by motion capture, pressure sensing device, and Kinect on two different days.

**Table 1:** Summary of the participants’ characteristics.

Participants	Sex	Height (cm)	Body weight (kg)
1	Male	163	53
2	Male	168	61
3	Male	176	62
4	Male	167	63
5	Male	183	70
6	Male	168	55
7	Male	175	61
8	Female	157	45
9	Male	164	65
10	Male	170	57

### Variables

Kinematic parameters including gait velocity, gait cycle time, step length, maximum and minimum flexion angle of the hip joint, maximum and minimum flexion angle of the knee joint, ground contact time of foot, and maximum ankle velocity were calculated by the methods as shown in Table 2.

**Table 2:** Identification method of all variables for Kinect and motion capture system.

Variables	Kinect	Motion capture
Gait speed (m/s)	Mean velocity of the spine base in the AP plane	Mean velocity calculated from the time and distance between one heel strike and the next heel strike, judged from the pressure sensing device
Gait cycle time (s)	Time between one heel strike and the next heel strike, judged from hip-knee cyclogram	The time between one heel strike and the next heel strike, judged from the pressure sensing device
Step length (m)	The length between one and another ankle in the AP plane	The length between one and another toe
Hip joint angle (degree)	The angle between the “hip-knee vector” and the “vertical axis” in the AP plane	The angle between the “hip-knee vector” and the “vertical axis” in the 3D coordinates axis, judged from motion capture
Knee joint angle (degree)	The angle between the “knee-hip vector” and the “knee-ankle vector” in the AP plane	
Ground contact time of foot (s)	The time of the “ankle velocity” in the AP plane exceeding a threshold of +1 m/s	Time between one heel strike and toe off, judged from pressure sensing device
Maximum ankle velocity (m/s)	The maximum velocity of the ankle in the AP plane	The maximum velocity of the ankle in the AP plane

AP: anteroposterior.

### Gait analysis procedures

**Figure 1:**
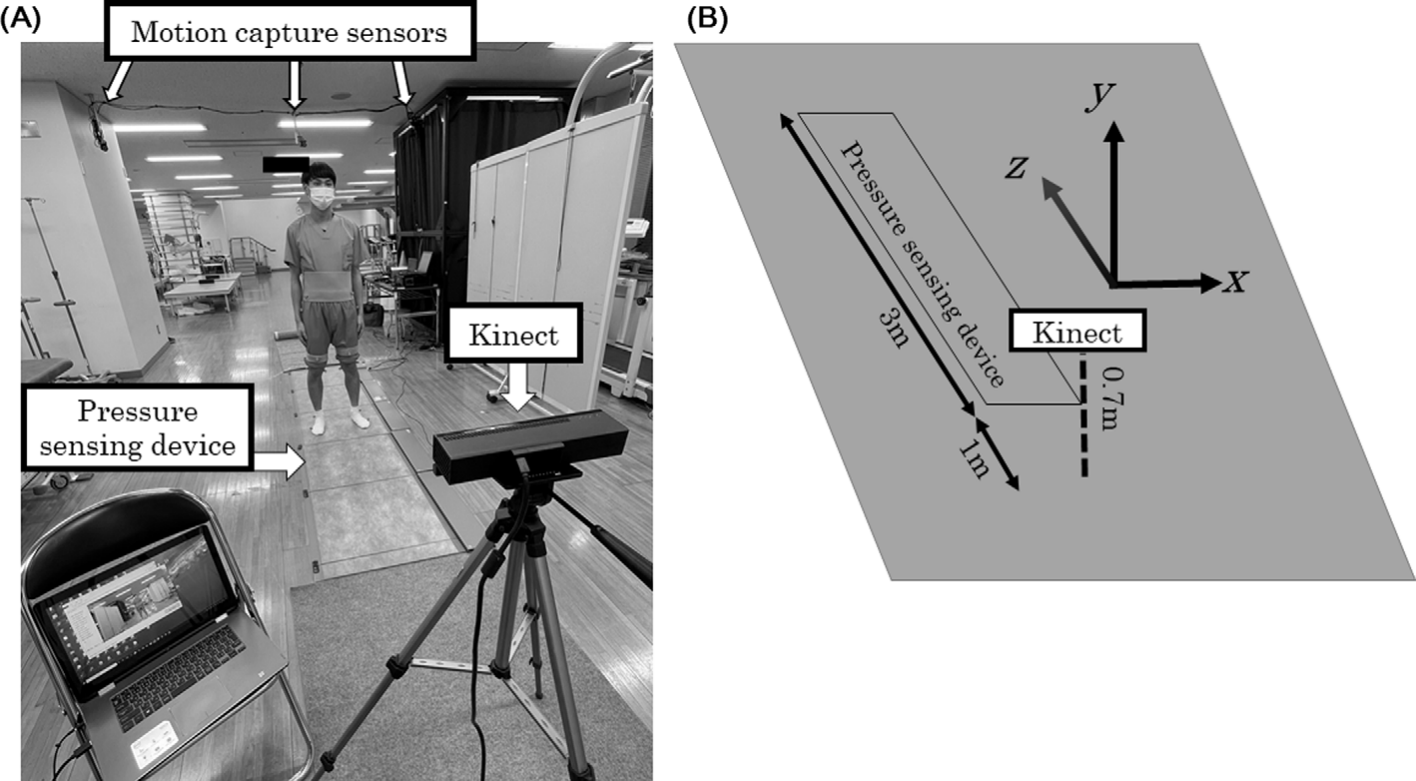
(A) Six infrared sensors were located around the participant. Single Kinect V2^®^ and a pressure sensing device were set in front of the participant. Participants walk from 4 m to 1 m on the plantar pressure sensing device towards Kinect. (B) The illustration shows the Kinect coordinate axis. *X*, *Y*, and *Z* directions show mediolateral, vertical, and posteroanterior axis, respectively.

Kinematic parameters were obtained by Kinect, infrared- and marker-based-motion capture system (MA-3000^®^; ANIMA Inc., Tokyo, Japan), and plantar pressure sensing device (Walk Way MV-1000^®^; ANIMA Inc.), simultaneously. Kinect was placed on a tripod about 70 cm in front of the participants. The participants were instructed to walk from 4 m to 1 m toward the Kinect (Figure 1A). Using the Kinect software development kit (SDK), the estimated joint position of the spine shoulder, spine mid, spine base, hip, knee, ankle, and foot were obtained at 30 Hz [8, 10]. The coordinates axis of Kinect was defined as follows: *X* was the mediolateral axis, *Y* was the vertical axis, and *Z* was the posteroanterior axis (Figure 1B). Because the Kinect was in front of the participant, the *Z*-axis corresponded to the walking direction.

Gait analysis by infrared- and marker based-motion capture system was performed as follows: The participants’ neck, acromion process, elbow, wrist, posterior superior iliac spine, anterior superior iliac spine, greater trochanter, lateral condyle of femur, lateral malleolus, and fourth metatarsal bone all had reflective markers attached to them. Six infrared sensors tracked each position. The sampling frequency was 100 Hz. A plantar pressure sensing device was used to analyze the walk cycle, step length, and ground contact time of the foot. The device, with a sheet length of 3 m, was installed in the middle of the walkway by connecting three modules as previously described [11]. The data obtained from both infrared- and marker-based-motion capture systems and the pressure sensing device were synchronized and output by the software (MD-1000^®^; ANIMA Inc.).

Each participant walked at least 5 times on different days. Every walk was captured by Kinect, at infrared- and marker-based-motion capture system, and pressure-sensing devices simultaneously. One walk cycle was defined as the period from one heel strike to the next.

Regarding the analysis by Kinect, we used the kinematic data from the *Y* and *Z*-axis to calculate variables. One walk cycle was extracted by using a hip-knee cyclogram, which shows the relationship between hip and knee joint angle [12]. This cyclogram allows for the estimation of the heel strike and the extraction of one walk cycle. The walk and foot speeds were calculated using the speed in the Z-axis of the spine base and the ankle, respectively. The maximum distance between the left and right ankles in the *Z*-axis was used to define step length. Two vectors (hip joint angle, hip-knee vector, and gravity vector) were used to calculate the joint angle. Knee joint angle; knee-hip vector and knee-ankle vector) in the sagittal plane (*Y*–*Z* plane). The ground contact time of the foot was defined as the period of ankle speed <1 m/s on the *Z*-axis.

### Statistical analysis

Each variable obtained by Kinect were compared to those by infrared- and marker-based-motion capture system and a pressure sensing device, respectively. Pearson’s correlation coefficients were calculated for each participant, and each correlation coefficient was then integrated using a meta-analysis method. Models for locally estimated scatterplot smoothing (LOESS). The forest plot was calculated using gait velocity, gait cycle time, and the maximum flexion angle of the hip and knee joints. All the analyses were conducted using the R statistics package, version 3.6.1 (R Core Team, Foundation for Statistical Computing, Vienna, Austria). The *P*-value < 0.05 was regarded as statistically significant.

## Results

The correlation coefficients for all parameters ranged from 0.22 to 0.92 (Table 3). The correlation coefficients were regarded as very strong reliability in gait velocity (*r* = 0.92, *p* < 0.01), step length (*r* = 0.81, *p* < 0.01). The correlation coefficients were regarded as moderate in gait cycle time (*r* = 0.65, *p* < 0.01), minimum flexion angle of hip joint (*r* = 0.68, *p* < 0.01). The correlation coefficients were regarded as fair in maximum flexion angle of hip joint (*r* = 0.43, *p* = 0.011), maximum flexion angle of knee joint (*r* = 0.54, *p* < 0.01). The correlation coefficients were regarded as poor in minimum flexion angle of knee joint (*r* = 0.23, *p* = 0.07), ground contact time of foot (*r* = 0.23, *p* = 0.08), and maximum ankle velocity (*r* = 0.22, *p* = 0.011) (Table 3) [13].

**Table 3:** Results of every variable, and Pearson’s correlation coefficients (*r*).

Variables	Correlation	Lower limit	Upper limit	*p* value
Gait velocity	0.92	0.87	0.95	*p* < 0.01
Gait cycle time	0.65	0.44	0.79	*p* < 0.01
Step length	0.81	0.73	0.87	*p* < 0.01
Maximum FA of hip joint	0.43	0.10	0.67	0.011
Minimum FA of hip joint	0.68	0.40	0.84	*p* < 0.01
Maximum FA of knee joint	0.54	0.36	0.68	*p* < 0.01
Minimum FA of knee joint	0.23	−0.02	0.45	0.07
Ground contact time of foot	0.23	−0.02	0.46	0.08
Maximum ankle velocity	0.22	0.05	0.38	0.011

FA: flexion angle.

**Figure 2:**
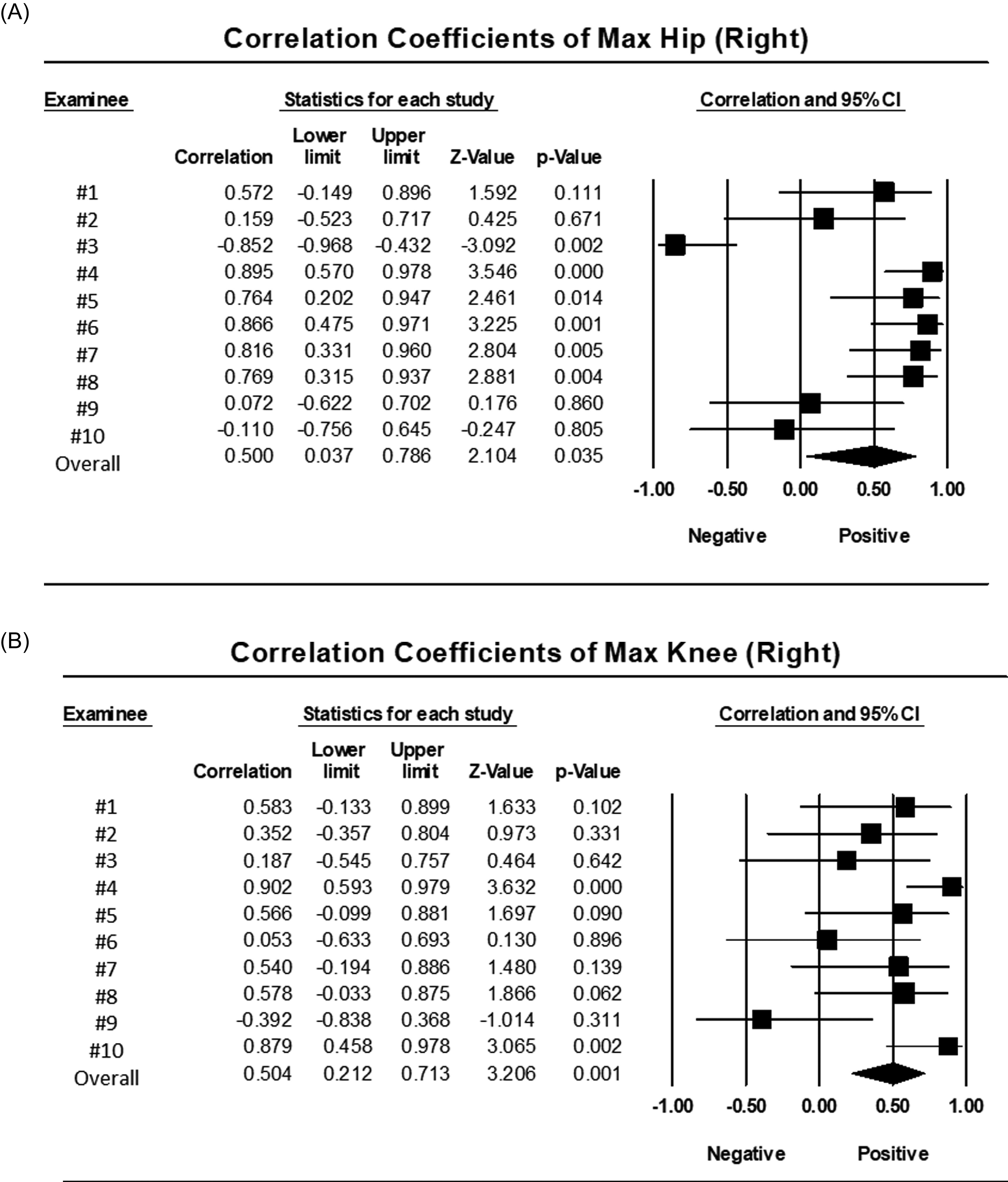
Correlation coefficients of maximum flexion angle of right hip (A) and right knee (B) in each subject varied among each subject. Overall meta-analysis shows that maximum flexion angle of hip and knee correlates with *r* = 0.500 and *p* = 0.035, and *r* = 0.504 and *p* < 0.001, respectively.

To investigate why correlation coefficients were low in minimum flexion angle of the knee joint, ground contact time of foot, and maximum ankle velocity, we further investigated correlation coefficients based on each subject in gait velocity, gait cycle time, and maximum flexion angle of hip and knee joints. As a result, the correlation coefficients in gait velocity and gait cycle time did not differ across subjects (Supplemental Figures S1A and S1B). The correlation coefficients of maximum flexion angle of hip and knee joints, on the other hand, varied (Figures 2A and 2B).

In addition, the analysis of individual subjects revealed that the participants who showed small hip and knee flexion angles tended to present poor correlations in maximum flexion angle of hip and knee joints in the Kinect group. Individual analysis revealed that participants (#8–10) had low correlation coefficients for hip and knee joint angles. These participants (#8–10) had a small average maximum flexion angle of the hip and knee joints, which was measured using an infrared and marker-based-motion capture system. Conversely, participants (#1, 4, 5, 7) showing the average maximum flexion angle of hip and knee joints more than 40° showed strong and fair correlation coefficients (Table 4).

**Table 4:** Degrees of the maximum flexion of hip and knee joint of each participant.

Participants	1	2	3	4	5	6	7	8	9	10
Maximum FA of hip joint (degree)	44.4 ± 25.2	36.6 ± 18.0	34.1 ± 20.6	41.2 ± 19.5	45.4 ± 21.0	39.7 ± 23.8	45.5 ± 22.2	21.8 ± 10.3	22.5 ± 7.6	12.8 ± 11.5
Maximum FA of knee joint (degree)	69.0 ± 4.6	64.3 ± 4.0	62.5 ± 3.2	61.9 ± 4.7	66.4 ± 3.0	65.4 ± 4.1	63.4 ± 4.2	58.1 ± 3.8	58.8 ± 2.7	54.5 ± 9.4

FA: flexion angle.

**Figure 3:**
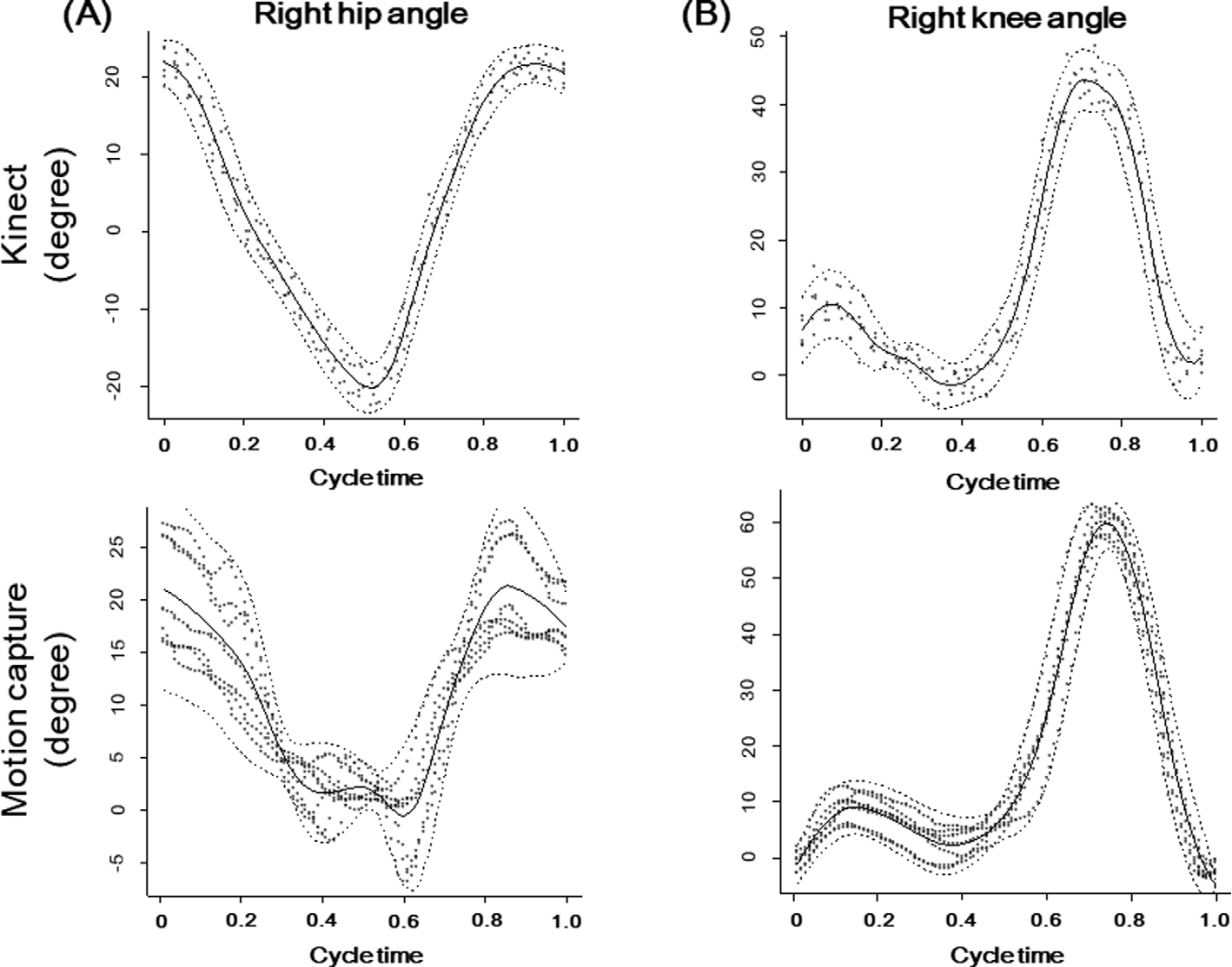
The LOESS models show right hip (A) and knee angle (B) of participant (#9) over one gait cycle. Solid lines, which express the average angle of all walks, have similar trajectories in Kinect and motion capture groups.

LOESS models were used to show hip and knee angles in the Kinect group and motion capture group, revealing that both shapes were similar to each other (Figure 3).

## Discussion

Measurement of gait parameters is indispensable for human gait analysis and useful to understand changes in joint function in orthopedic surgery. In this study, we investigated whether Kinect, a peripheral device of Xbox^®^ that acquires information such as depth, posture, and skeleton definition, can be used for human gait analysis in healthy subjects. We found that gait parameters, including gait velocity, gait cycle time, step length, minimum flexion angle of the hip joint, and maximum flexion angle of the knee joint obtained by Kinect, were well correlated with those by infrared- and marker based-motion capture system.

There were some limitations in this study. The sample size was small. To consider the measurement errors, we collected at least 10 walks for each participant on different days in this study. Furthermore, we matched the target walking cycle between the Kinect and motion capture groups. Because the majority of the participants in this study were young men, the kinematic variables may have been skewed. Taking our findings into account, the kinematic variables examined in this study would not differ based on gender.

The usage of Kinect in gait analysis has not been fully established. Numerous studies on human gait analysis using the infrared- and marker-based-motion capture system have been reported in the field of orthopedic surgery [7–9]. In general, the infrared- and marker-based-motion capture system requires a lot of markers attached to the body and performs gait analysis [7–9]. Although Kinect lacks markers, proper gait analysis parameters were obtained in this study. Thus, it is likely that Kinect could be used as an Xbox^®^ peripheral device and as a gait analysis device in orthopedic surgery. It also appears that Kinect does not require a fixed-point camera and can easily perform gait analysis without the use of marker settings.

As for the methods of gait analysis, a treadmill walk survey shows that Kinect enables to measure hip and knee joint trajectories in healthy subjects [14]. It has also been reported that the Kinect accurately captures hip and knee joint trajectories but not ankle joint trajectories [15] (Table 5). Although gait analysis on a treadmill is useful because it can be performed in a small space with healthy subjects, it appears difficult for patients with severe musculoskeletal disease such as osteoarthritis. Thus, our method that participants walk a few meters on a flat surface seemed to be simple and effective even for patients with joint disease. On the other hand, walking speed or step length would be the good kinematic variables reflecting gait impairments caused by joint disease [3, 4]. Previous studies reported that gait parameters, including gait velocity, gait cycle time, and step length, obtained by Kinect are reliable [8, 10]. The values of gait velocity, gait cycle time, and step length in this study were similar to those in previous papers [8, 10]. As a result, Kinect may be a tool for accurately measuring kinematic variables that reflect gait impairments caused by joint disease in the field of orthopedic surgery.

**Table 5:** Study, participants’ characteristics and gait analysis devices.

Author (year)	Number (gender)	Gait analysis device	Standard motion capture device	Methods
Usami et al. (2022)	*N* = 10 (9 males, 1 female)	One Kinect V2	MA-3000 and Planter Walk Way MV-1000	Walking on flat ground from 4 m
Geerse et al. [8]	*N* = 21 (11 males, 10 females)	Four Kinect V2	Optotrak	Walking on flat ground from 10 m
Xu et al. [14]	*N* = 20 (10 males, 10 females)	One Kinect V2	Optotrak	Walking on treadmill
Timmi et al. [15]	*N* = 20 (11 males, 9 females)	One Kinect V2	VICON	Walking on treadmill
Guffanti et al. [16]	*N* = 6 (Gender undescribed)	Two Kinect V2	VICON	Walking on flat ground from 4.8 m

Regarding the hardware and software, the mechanism of posture recognition is based on projecting a pulsed infrared pattern and simultaneously measuring the reflection with an infrared sensor and a red/green/black (RGB) sensor. Kinect measures the time of flight (TOF) for each pattern and then matches the data to the RGB image [16]. Our preliminary study showed that the detection of heel strike judged from the position of the ankle was not precisely recognized because infrared was blocked in the case where the forefoot covers the ankle. Thus, to recognize exact ankle detection, we adopted a hip-knee cyclogram in this study [12]. As for the number of Kinect installed for gait analysis, multiple Kinect systems have been used for gait analysis [8, 16] (Table 5). However, the use of multiple Kinect systems requires relatively high cost and technical skills to integrate each data. In this study, we used a single Kinect sensor to capture gait variables, eliminating the need for data integration. Latorre et al. used a single Kinect sensor to assess gait parameters such as walking speed and step length. The study, however, lacked a standard system as a control [17]. We compared each variable between Kinect and the motion capture system and found that Kinect showed a good correlation with the motion capture system. On the other hand, we examined correlation coefficients in gait velocity, gait cycle time, and maximum flexion angle of hip and knee joints based on each subject and discovered that the correlation coefficients of maximum flexion angle of hip and knee joints varied from each subject, whereas correlation coefficients in gait velocity and gait cycle time did not differ in all participants. In addition, the analysis of individual subjects revealed that the participants who showed small hip and knee flexion angles under 40° tended to present poor correlations in maximum flexion angle of hip and knee joints. Taking these findings into account, it is most likely that kinematic and spatiotemporal parameters obtained by Kinect in participants with a large bending angle of the hip and knee joints are reliable. Further investigation will be necessary to evaluate these gait parameters in patients with hip or knee joint disorders.

In conclusion, our results strongly suggest that Kinect could be a reliable device in lower extremity gait analysis compared to the infrared- and marker-based-motion capture system.

## Figures and Tables

**Figure S1A:**
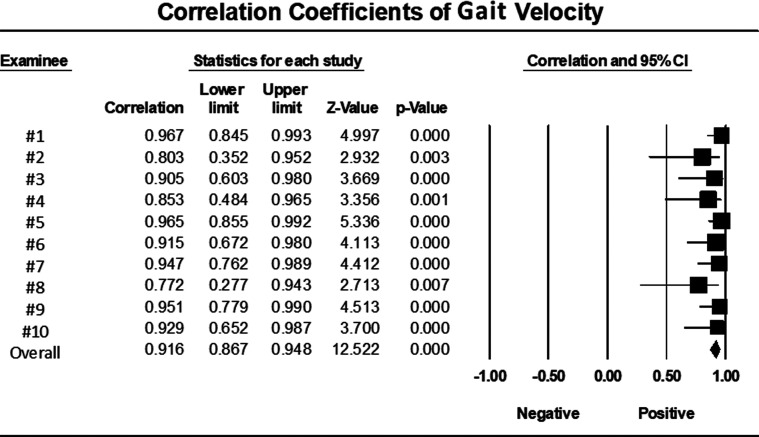
Correlation coefficients of gait velocity in each subject, respectively.

**Figure S1B:**
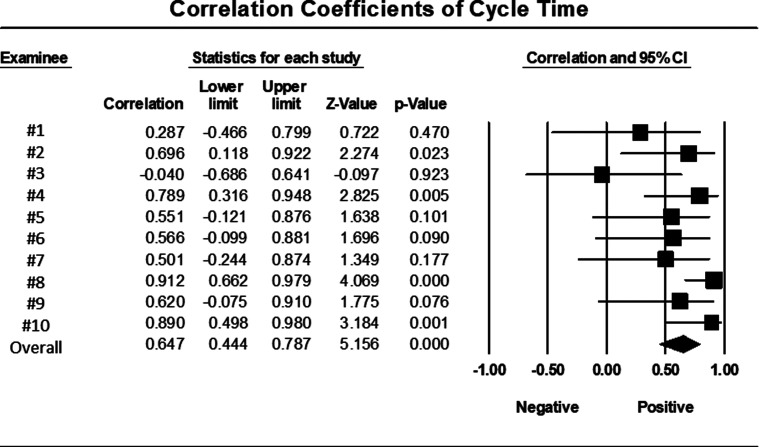
Correlation coefficients of gait cycle time in each subject, respectively.
